# Proton and carbon ion beam treatment with active raster scanning method in 147 patients with skull base chordoma at the Heidelberg Ion Beam Therapy Center—a single-center experience

**DOI:** 10.1007/s00066-022-02002-4

**Published:** 2022-09-23

**Authors:** Matthias Mattke, Matteo Ohlinger, Nina Bougatf, Semi Harrabi, Robert Wolf, Katharina Seidensaal, Thomas Welzel, Falk Röder, Sabine Gerum, Malte Ellerbrock, Oliver Jäkel, Thomas Haberer, Klaus Herfarth, Matthias Uhl, Jürgen Debus

**Affiliations:** 1 Department of Radiation Oncology, Paracelsus Medical University, SALK, Salzburg, Austria; 2 Department of Radiation Oncology, Heidelberg University Hospital, Heidelberg, Germany; 3 Heidelberg Institute of Radiation Oncology (HIRO), Heidelberg, Germany; 4 National Center for Tumor diseases (NCT), Heidelberg, Germany; 5 Clinical Cooperation Unit Radiation Oncology, German Cancer Research Center (DKFZ), Heidelberg, Germany; 6 Heidelberg Ion-Beam Therapy Center (HIT), Department of Radiation Oncology, Heidelberg University Hospital, Heidelberg, Germany; 7 German Cancer Consortium (DKTK), Heidelberg, Germany; 8 Department of Radiation Oncology, Ludwigshafen Hospital, Bremserstr. 79, 67063 Ludwigshafen, Germany; 9 Müllner Hauptstr. 48, 5020 Salzburg, Austria

**Keywords:** Notochordal sarcoma, Particle therapy, Heavy ion, High LET, Bragg peak, C12

## Abstract

**Background:**

This study aimed to compare the results of irradiation with protons versus irradiation with carbon ions in a raster scan technique in patients with skull base chordomas and to identify risk factors that may compromise treatment results.

**Methods:**

A total of 147 patients (85 men, 62 women) were irradiated with carbon ions (111 patients) or protons (36 patients) with a median dose of 66 Gy (RBE (Relative biological effectiveness); carbon ions) in 4 weeks or 74 Gy (RBE; protons) in 7 weeks at the Heidelberg Ion Beam Therapy Center (HIT) in Heidelberg, Germany. The median follow-up time was 49.3 months. All patients had gross residual disease at the beginning of RT. Compression of the brainstem was present in 38%, contact without compression in 18%, and no contact but less than 3 mm distance in 16%. Local control and overall survival were evaluated using the Kaplan–Meier Method based on scheduled treatment (protons vs. carbon ions) and compared via the log rank test. Subgroup analyses were performed to identify possible prognostic factors.

**Results:**

During the follow-up, 41 patients (27.9%) developed a local recurrence. The median follow-up time was 49.3 months (95% CI: 40.8–53.8; reverse Kaplan–Meier median follow-up time 56.3 months, 95% CI: 51.9–60.7). No significant differences between protons and carbon ions were observed regarding LC, OS, or overall toxicity. The 1‑year, 3‑year, and 5‑year LC rates were 97%, 80%, and 61% (protons) and 96%, 80%, and 65% (carbon ions), respectively. The corresponding OS rates were 100%, 92%, and 92% (protons) and 99%, 91%, and 83% (carbon ions). No significant prognostic factors for LC or OS could be determined regarding the whole cohort; however, a significantly improved LC could be observed if the tumor was > 3 mm distant from the brainstem in patients presenting in a primary situation.

**Conclusion:**

Outcomes of proton and carbon ion treatment of skull base chordomas seem similar regarding tumor control, survival, and toxicity. Close proximity to the brainstem might be a negative prognostic factor, at least in patients presenting in a primary situation.

**Supplementary Information:**

The online version of this article (10.1007/s00066-022-02002-4) contains supplementary material, which is available to authorized users.

## Introduction

Chordomas are rare malignant bone tumors that most likely arise from remaining cells of notochordal development. Thus, the most common locations are along the neuroaxis [1]. According to the Surveillance, Epidemiology, and End Results (SEER) database, the overall incidence is 8.4 per 10 million [2]. To date, there are no known risk factors. Usually, chordomas are locally and aggressively growing low-grade tumors that are prone to high local recurrence rates [2, 3]. The pathological classification differentiates between classic, chondroid, and dedifferentiated subtypes. Subtype is associated with prognosis, with the chondroid subtype having the best and the dedifferentiated subtype the worst [4–6]. Because of the low potential for metastasis [7, 8], the most important prognostic factor in treatment is achievement of local control (LC) [9]. Due to their typical location with critical structures of the skull base in close proximity, complete surgical removal is often impossible. Hence, macroscopic residual tumor is often found even after attempted complete resection and may result in insufficient tumor control and a worse overall outcome [10]. Therefore, function-preserving surgery followed by (proton) radiotherapy is currently seen as the gold standard instead of radical surgery alone. However, dose escalation to at least 70 Gy (relative biological effectiveness, RBE) seems necessary due to the low radiosensitivity of chordomas [11], which is often difficult to achieve due to directly adjacent vital structures. Therefore, highly conformal radiation techniques like proton or carbon ion beam therapy appear to be favorable [12]. For example, Uhl et al. found promising local control rates of 72% after 5 years using carbon ions [13]. Moreover, carbon ions have a higher RBE than photons or protons, which may increase treatment efficacy compared to protons [14], although this has not been validated in a clinical study. The purpose of this study was to compare carbon ions to protons in the treatment of the skull base. In addition, multiple factors such as proximity of the tumor to organs at risk were evaluated concerning a possible influence on oncological results.

## Materials and methods

All patients with classic and chondroid skull base chordomas who were treated at the Heidelberg Ion Beam Therapy Center (HIT) between 2009 and 2014 outside the randomized trial were retrospectively included into the analysis. To achieve a definitive demarcation from chondrosarcoma, a test for expression of Brachyury was performed at our center if the samples hadn’t already been tested by the referring hospital [15]. Reference pathology was not obtained routinely.

We aimed to generate a homogenous collective. Therefore, patients with dedifferentiated chordoma were excluded due to their worse prognosis and their small number in the overall collective (total of 3 patients). Patients younger than 18 years or patients who had been previously irradiated to the skull base were also excluded. No further patient selection for the analysis was performed (particularly, no treated patient was excluded from analysis due to comorbidity, high age, or other risk factors).

### Pretreatment procedures and patient characteristics

All patients received MRI of the skull base for treatment planning and at least a thoracic CT scan for staging purposes. Patients were discussed at a multidisciplinary tumor board (including an experienced neurosurgeon) prior to radiation treatment. Detailed information on patient characteristics can be found in Table 1.

**Table 1:** Patient characteristics, LC, and OS rates

	All	C12	H1
Number of patients	147	111	36
Men/women	57.8% (85)/42.2% (62)	56.8% (63)/43.2% (48)	61.1% (22)/38.9% (14)
Primary/recurrent	76.9% (113)/23.1% (34)	76.6% (85)/23.4% (26)	77.8% (28)/22.2% (8)
Median age at radiation	51 years	51 years	50 years
Median boost volume (CTV)	40.4 ml	40.9 ml	38.3 ml
Median follow-up time	49.3 months	52.2 months	36.5 months
LC rates at 1, 3, 5 years	96.2%, 80.5%, 63.7%	96.1%, 80.4%, 64.5%	96.7%, 79.8%, 60.7%
OS rates at 1, 3, 5 years	99.3%, 91.4%, 84.9%	99.0%, 91.2%, 83.3%	100%, 91.7%, 91.7%

*CTV* clinical target volume, *LC* local control, *OS* overall survival

### Treatment planning and delivery

Patients were immobilized using a thermoplastic head mask system. All patients received computed tomography (3-mm slice thickness) for three-dimensional treatment planning. For exact contouring of the treatment volume and organs at risk, a three-dimensional correlation with contrast-enhanced T1-weighted and T2-weighted (T2-STIR) magnetic resonance imaging (MRI) was performed, which was then rigidly registered on the CT scan. All patients had macroscopic residual disease. Compression of the brainstem was present in 38%, contact without compression in 18%, and no contact but less than 3 mm distance in 16%.

For the boost clinical target volume (CTV2), a margin of 1–2 mm was applied to the gross tumor volume (GTV). The primary clinical target volume (CTV1) contained CTV2 and the preoperative tumor extent. Both PTVs (PTV1 and PTV2) were formed by adding a safety margin of 3 mm to the corresponding CTV. The Siemens Syngo PT Planning software (Siemens Healthineers, Erlangen, Germany) was used for treatment planning. The treatment was performed at the HIT in Heidelberg, Germany, using protons and carbon ions in active raster scan technique. Dose distribution was calculated using the local effect model 2 (LEM2). Dose constraints to OARs were prescribed according to the QUANTEC database, particularly the brainstem was limited to a maximal dose of 59 Gy (RBE; EQD2) [16]. If dose prescription goals and dose constraints could not be met at the same time, underdosage of the target was preferred. Detailed information concerning carbon ion treatment has been published previously [17].

A total of 111 patients received carbon ion treatment (C12) with a median total dose of 66 Gy (RBE; 45 Gy to PTV1, 21 Gy to PTV2) in a fractional dose of 3 Gy (RBE) six times per week (treatment on Saturdays). The remaining 36 patients received proton treatment (H1) with a median total dose of 74 Gy (RBE; 50 Gy to PTV1 and 24 Gy to PTV2) in 2‑Gy (RBE) fractional doses six times per week. If the total median dose of hypofractionated carbon ion therapy is calculated in EQD2 with an alpha/beta value of 10, the total dose is 71.5 Gy, and thus comparable to the proton dose of 74 Gy (RBE).

The decision regarding the type of radiation quality used was triggered by in-house standards. During the first years of the HIT, patients received carbon ion treatment as our standard therapy based on our previously published experiences from GSI (where no protons had been available) [18]. With growing experience in proton therapy, this practice was changed in 2013 to the worldwide standard of care using protons. Since then, carbon ion therapy has only been offered to patients in a prospective randomized trial. This also explains the longer follow-up time for carbon ions. No patient received chemotherapy.

### Follow-up

Patients were scheduled for regular follow-up visits including MRI of the skull base every 3 months for the first 2 years and on an individual basis concerning the intervals thereafter. Because of the long travel distances, virtual follow-up examinations with in-house review of outpatient MRI imaging and telephone calls for symptom assessment were possible based on the individual patient preference. In addition, all patients were mailed with an information sheet and a questionnaire regarding the current tumor status and possible side effects. All data were collected in the central HIRO research database at the authors’ department, as has been described previously [19]. Because of the retrospective character of the prevailing analysis, the documentation of symptoms at baseline and during follow-up was not standardized. The symptoms in the questionnaire were graded according to the CTCAE manual. Staging procedures regarding distant failure during follow-up were at the discretion of the referring center, but data were included into the current analysis if available.

### Statistical analysis and legal considerations

All medical records and follow-up MRI scans were evaluated retrospectively. LC was defined as time from the first day of radiation treatment until local progression, OS was defined as time from the first day of radiation treatment until death. Distant failure rate or CSS was not assessed due to inconsistent (distant) follow-up procedures and low autopsy rates (see above). Patients without events were censored at the time of the last follow-up. Descriptive data were reported using median and range, LC and OS were reported using the Kaplan–Meier method. Subgroups were compared using the log-rank test. A *p*-value ≤ 0.05 was defined as statistically significant. The current study was approved by the independent ethics committee at the University of Heidelberg.

## Results

### Local control, overall survival, and prognostic factors

A total of 41 patients (27.9%) developed a local recurrence during the follow-up period. Local control in all patients at 1, 3, and 5 years was 96%, 81%, and 64%, respectively. Overall survival at 1, 3, and 5 years was 99%, 91%, and 85%, respectively (Graphs 3, 4; supplement).

**Fig. 1:**
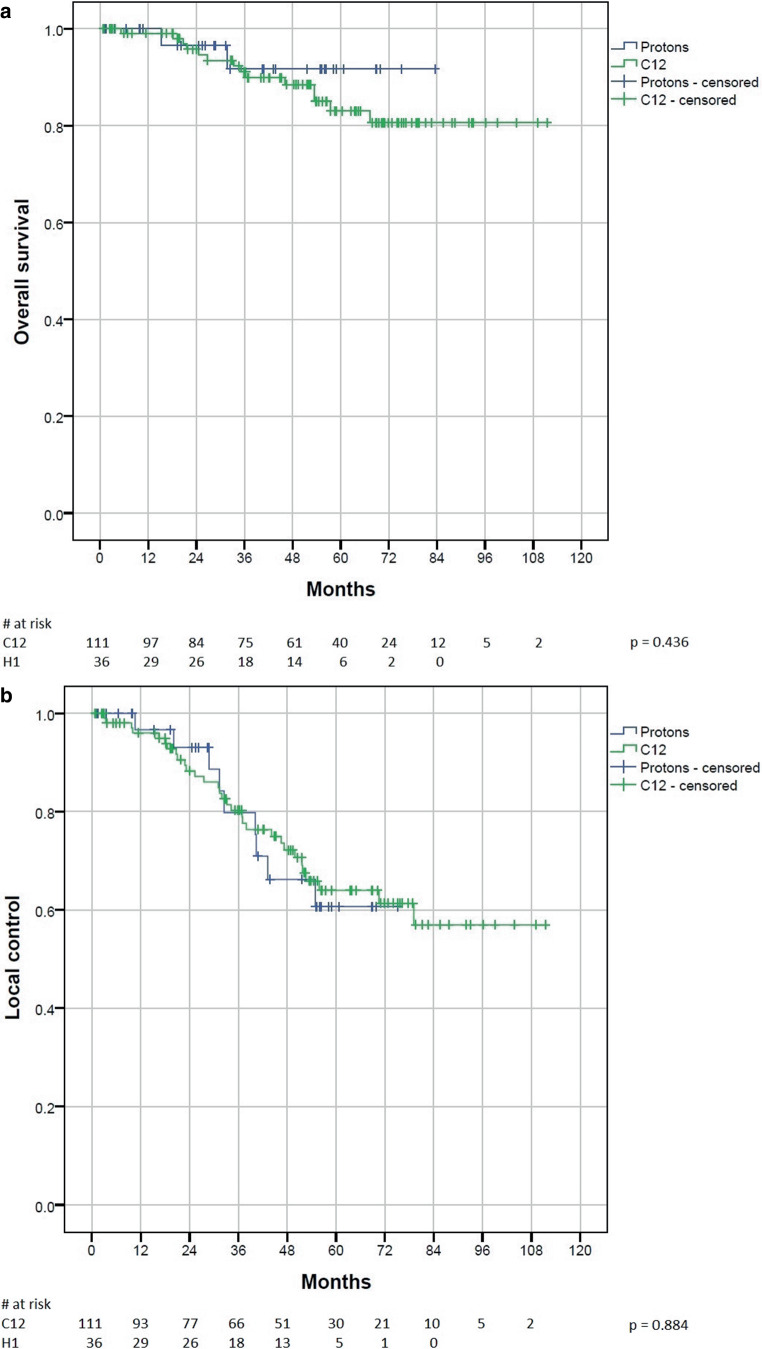
**a** Overall survival and **b** local control compared between protons and carbon ions

The 1‑year, 3‑year, and 5‑year LC rates were 97%, 80%, and 61% for protons and 96%, 80%, and 65% for carbon ions, respectively (Fig. 1a). The OS rates in the same periods of time were 100%, 92%, and 92% for protons and 99%, 91%, and 83% for carbon ions, respectively (Fig. 1b).

No statistically significant difference between protons and carbon ions could be determined, neither regarding LC (*p* = 0.87) nor concerning OS (*p* = 0.45).

**Fig. 2:**
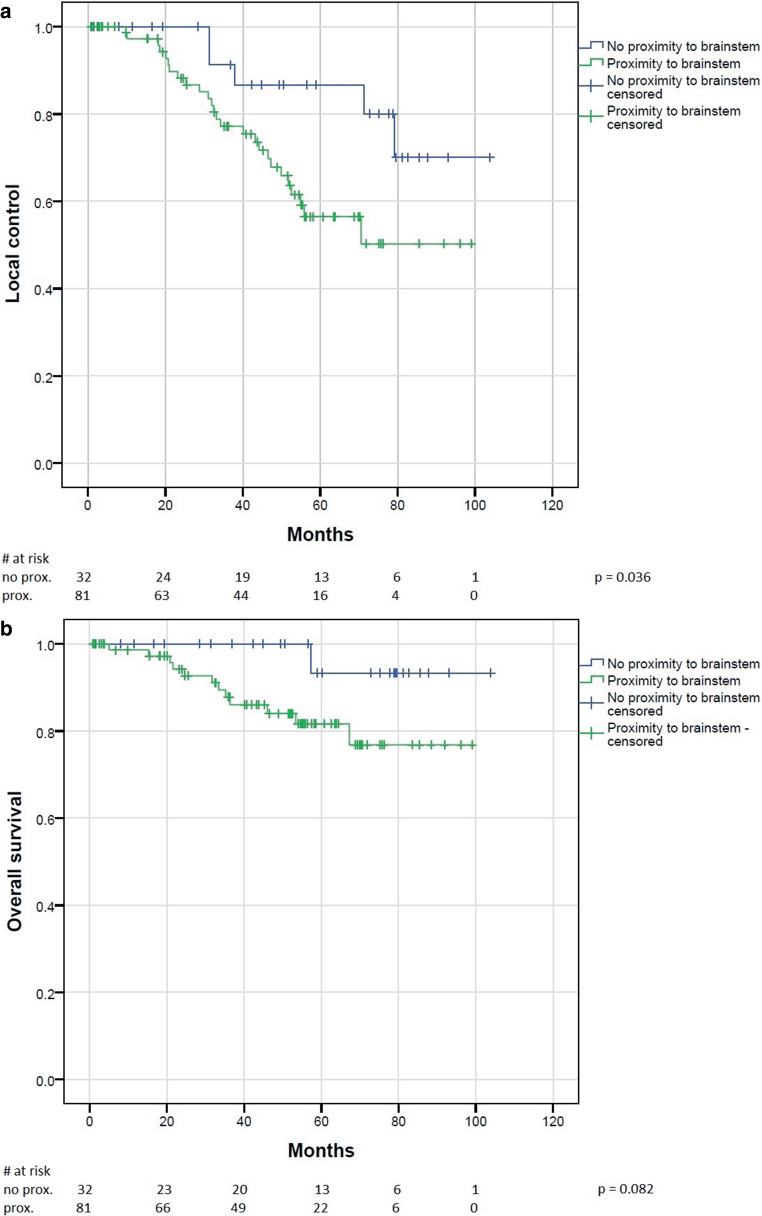
**a** Local control and **b** overall survival in a primary situation dependent on the proximity of the tumor to the brainstem

Subgroup analyses revealed a trend towards better LC in men in the current analysis (*p* = 0.06). No significant differences regarding LC were found based on patient age (*p* = 0.44; Graphs 5, 6; supplement) and none of the mentioned factors were significantly associated with overall survival. Regarding primary versus recurrent situation and proximity to brainstem, again no significant differences in LC or OS were observed regarding the whole cohort (Graph 7; supplement). However, if primary and recurrent tumors were analyzed separately, we found a significantly improved LC rate (*p* = 0.036) and a trend toward improved OS (*p* = 0.082) in primary patients with tumors more than 3 mm distant from the brainstem (Fig. 2a, b), while no significant differences in LC (*p* = 0.318) or OS (*p* = 0.326) were present in the recurrent subgroup.

### Toxicity

Acute side effects like mucositis or skin toxicity were generally mild (none > CTC grade II) and similar between carbon ions and protons. A total of 44 patients with temporal lobe reactions were identified. Among these, the most severe late side effect was temporal lobe necrosis found in 20 patients, of which most cases were asymptomatic (CTC grade I) or responded well to therapy with steroids or bevacizumab (CTC grade II–III). No significant difference regarding temporal reactions (including necrosis) between protons (*n* = 11; 31%) and carbon ions (*n* = 33; 29.8%) could be determined. A detailed analysis of late toxicity with special regard to the association of temporal lobe necrosis with dosimetric factors is currently in preparation and will be published separately. Overall, no grade IV acute or late toxicity or treatment-related deaths were observed.

## Discussion

### Comparison to previously published results for carbon ion, proton, and photon irradiation

Herein, we present a retrospective comparison of carbon ion and proton treatments with regards to local control and overall survival. To our knowledge, our cohort represents one of the largest published single-center experiences reporting exclusively chordomas treated solely with particles. Moreover, all tumors were tested for the marker Brachyury to clearly distinguish between chordomas and chondrosarcomas and to exclude the latter [20].

Complete surgical removal of the tumor (with or without adjuvant RT) is the first choice of treatment but is often impossible due to the close proximity of vital structures. In case of unresectability or residual gross disease, radiotherapy in its different variants represents the only curative-intent treatment option.

Data concerning photon-only radiotherapy in skull base chordoma are scarce. The greatest difficulty with photons is the application of a sufficient dose due to lower radiation tolerance of the surrounding OARs. Forsyth et al. reported on a collective of 39 patients who received irradiation with a median dose of 50 Gy after surgery. The LC after 5 years was 39% [21]. Even with higher conformal photon techniques like a Gamma Knife, sufficient LC seems to be difficult to achieve. Debus et al. reported on local control rates of 82% after 2 and 50% at 5 years using stereotactic fractionated radiotherapy with a median dose of 66.6 Gy at the isocenter [22]. In the collective of Cho et al., seven patients were irradiated with a Gamma Knife after surgery. Four of those patients (57%) suffered from a relapse during the follow-up [23]. A recent analysis of 93 patients with small intracranial chordoma who underwent single-session SRS has been published by Pikis et al. [24] With a mean maximum dose of 34.2 Gy, the LC after 5 years was 54.7%. Notably, the irradiation volume was much smaller than in the prevailing study (mean 8 cc vs. 40.4 cc).

Because of these limitations, combination approaches of photons with particle boosts have been established. The largest cohorts published to date used proton/photon combinations [25, 26], reporting 5‑year LC rates of 73–75%. However, the first study was only presented as an abstract, giving no detailed information, e.g., regarding patient characteristics, while the second study included some patients after complete resection and is therefore difficult to compare. Because of the superior dose distribution of particles compared to photons, several institutions use pure proton or carbon ion treatments instead and observe 5‑year local control rates of 71–92% (including the results of our precursor facility, GSI). In the current study, we found a 5-year local control rate of 64%, which seems slightly inferior, although interstudy comparisons should be interpreted with extreme caution. Concerning this difference, two major points have to be taken into account: except for the exclusion of three patients with dedifferentiated chordomas, no dedicated patient selection concerning inclusion in the analysis (e.g., according to lesion size, adherence or compression to vital structures, comorbidities, or performance status) was performed. Moreover, all tumor specimens were tested for Brachyury, a marker highly specific for chordomas, which nearly eliminates the risk of inadvertent inclusion of chondrosarcomas with a lower risk for local recurrence after particle treatment.

We did not observe a significant difference in local control, overall survival, or toxicity based on the kind of treatment (protons vs. carbon ions). To the best of our knowledge, Iannalfi et al. have published the only prospective data on proton vs. carbon ion treatment of skull base chordomas [27]. They observed 5‑year local control rates of 84% with protons and 71% with carbon ions, but stated that unfavorable patients were specifically allocated to carbon ions. Consequently, the treatment arms were imbalanced with regard to GTV volume, quality of resection, primary versus recurrent disease, and deficits at baseline. Moreover, also patients after macroscopic complete resection were eligible and patients had to be in an adequate performance status. In contrast, all our patients had gross residual disease and no dedicated patient selection in general or regarding the treatment arm was performed. Thus, based on the limited available data, no clear advantage of either protons or carbon ions in the treatment of skull base chordomas can be concluded, at least considering the used dose schedules. In this context, it needs to be mentioned that there are still uncertainties regarding the accuracy of the RBE model (LEM) used for carbon ions [28]. The calculations are based on an RBE of 3–5 for C12 and 1.1 for H1. Especially concerning the newer results of proton therapy, there is a possibility that the estimation of the RBE of C12 may be too high and/or the estimation for H1 too low. Each combination would result in either an underdosing for C12 or an overdosing for H1. This remains subject to discussion and requires further investigation in the future.

### Major series reporting 5-year local control rates

Major series reporting 5‑year local control rates are presented in Table 2.

**Table 2:** Comparison of different studies concerning dose and local control [13, 25–27, 29–32]

Study	Modality	Patients (*n*)	Dose (Gy RBE)	LC rates (%)
Munzenrider et al. [25]	Photons + protons	169	66–83	5‑year: 73 10-year: 54
Fung et al. [26]	Photons + protons	106	68.4–73.8	2‑year: 88.6 4‑year:78.3 5‑year: 75.1
Weber et al. [29]	H1	151	74	5‑year: 75.8 7‑year: 70.9
Mizoe et al. [30]	C12	33	48–60.8	5‑year: 85 10-year: 64
Uhl et al. [13]^a^	C12	155	60 (57–70)	3‑year: 82 5‑year: 72 10-year: 54
Takagi et al. [31]	C12	13	57.6–74	5‑year: 92
Guan et al. [32]	H1 + C12	91	C12: 63–69	2‑year: 75.6
			H1: 70	2‑year: 100
			H1 + C12: 57–69	2‑year: 74.2
Iannalfi et al. [27]	C12/H1	65	C12: 70.4	3‑year: 77 5‑year: 71
			H1: 74	3‑year: 89 5‑year: 84
Current study	C12/H1	147	C12: 66	1‑year: 96 3‑year: 80 5‑year: 65
			H1: 74	1‑year: 97 3‑year: 80 5‑year: 61

^a^No overlap in patients with the prevailing analysis

*LC* local control, *RBE* relative biological effectiveness

### Proximity to organs at risk as a prognostic factor

The treatment of skull base chordoma is particularly difficult because of the proximity to a number of organs at risk. Especially relevant are the optic system, with visual impairments as a side effect, and the brainstem as the center of vital functions, which need to be preserved at all cost. Depending on the proximity of the tumor to these organs, especially the brainstem, a local underdosage of the tumor with a possibly higher risk of recurrence has to be accepted, probably resulting in inferior local control. Increasing evidence for this suggestion has emerged in recent years. For example, Takagi et al. reported on 24 patients treated with protons or carbon ions and observed a marked reduction in 5‑year local control (81% vs. 100%) in patients with close proximity of tumor and brainstem, although this difference was not statistically significant. Guan et al. reported on 91 patients with skull base or cervical chordomas treated with protons, carbon ions, or combinations of both, and observed significantly reduced LC, PFS, and OS in patients with compression of brainstem or the optic apparatus [32]. Weber et al. found worse local control and overall survival in a mixed cohort of skull base chordomas and chondrosarcomas if tumor compressed the brainstem or optic apparatus [29] and Iannalfi et al. reported similar results in their prospective trial using protons or carbon ions for skull base chordomas [27]. These findings are augmented by a recent analysis of Basler et al., who saw the majority of recurrences in the area of the brainstem in a mixed collective of chordoma and chondrosarcoma and were able to show a compromised dose distribution in those areas in their dosimetric analysis [33].

In our cohort, we strictly applied our predefined dose constraints especially for brainstem (59 Gy EQD2 based on the QUANTEC data), accepting possible target volume dose restrictions. Consistently, we found significantly inferior local control and a trend toward inferior survival in patients with tumors located less than 3 mm from the brainstem, if treated in a primary situation. Surprisingly, no marked differences were observed in patients treated in a recurrent situation, although this might be driven simply by the much smaller sample size of recurrent cases.

Our study has some limitations. As a retrospective single-center experience, some form of bias seems likely. Staging and follow-up procedures were less standardized than in prospective trials, especially regarding distant staging procedures, documentation of side effects, and causes of death. Moreover, efficacy comparisons of proton and carbon ion treatments generally inherit some uncertainties based on the necessarily used assumptions within the dosimetric calculation models (LEM2, RBE). Finally, the current report lacks a specific dosimetric analysis with regard to dose coverage in close proximity to critical organs at risk, which is part of an ongoing project and will be published separately.

## Conclusion

Carbon ion and proton therapy are effective treatments for patients with chordomas of the skull base concerning LC and OS. No significant differences with regard to outcome between the two modalities could be determined in this retrospective analysis. A randomized phase III superiority trial is currently investigating possible advantages of the carbon ion (60–66 Gy RBE) treatment compared to protons (70–76 Gy RBE) in the therapy of skull base chordoma and is still recruiting patients (clinicaltrials.gov identifier: NCT01182779). Proximity to the brainstem seems to be an unfavorable prognostic factor regarding local control, at least in the primary situation.

## Supplementary Information

Graph 4: Overall survival in all patients

Graph 3: Local control in all patients

Graph 5: Subgroup analysis concerning sex

Graph 6: Subgroup analysis concerning age

Graph 7: Subgroup analysis concerning primary vs. recurrent radiotherapy
