# Supplementary figures and images for: Proton and carbon ion beam treatment with active raster scanning method in 147 patients with skull base chordoma at the Heidelberg Ion Beam Therapy Center—a single-center experience
Source: Strahlenther Onkol. 2022 Sep 23;199(2):160–8. doi: 10.1007/s00066-022-02002-4 (PMC9876873; doi:10.1007/s00066-022-02002-4)

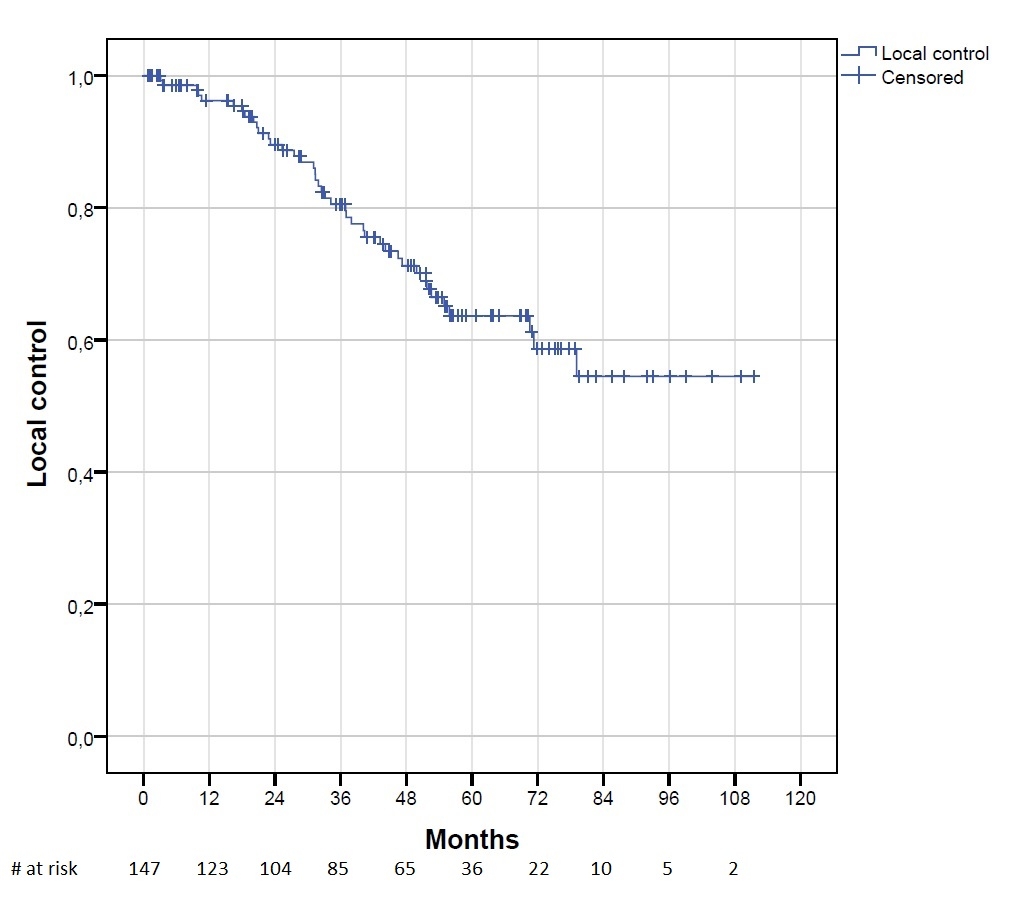

Supplement: Supplementary file 1 — Graph 4: Overall survival in all patients [file 66_2022_2002_MOESM1_ESM.jpg]

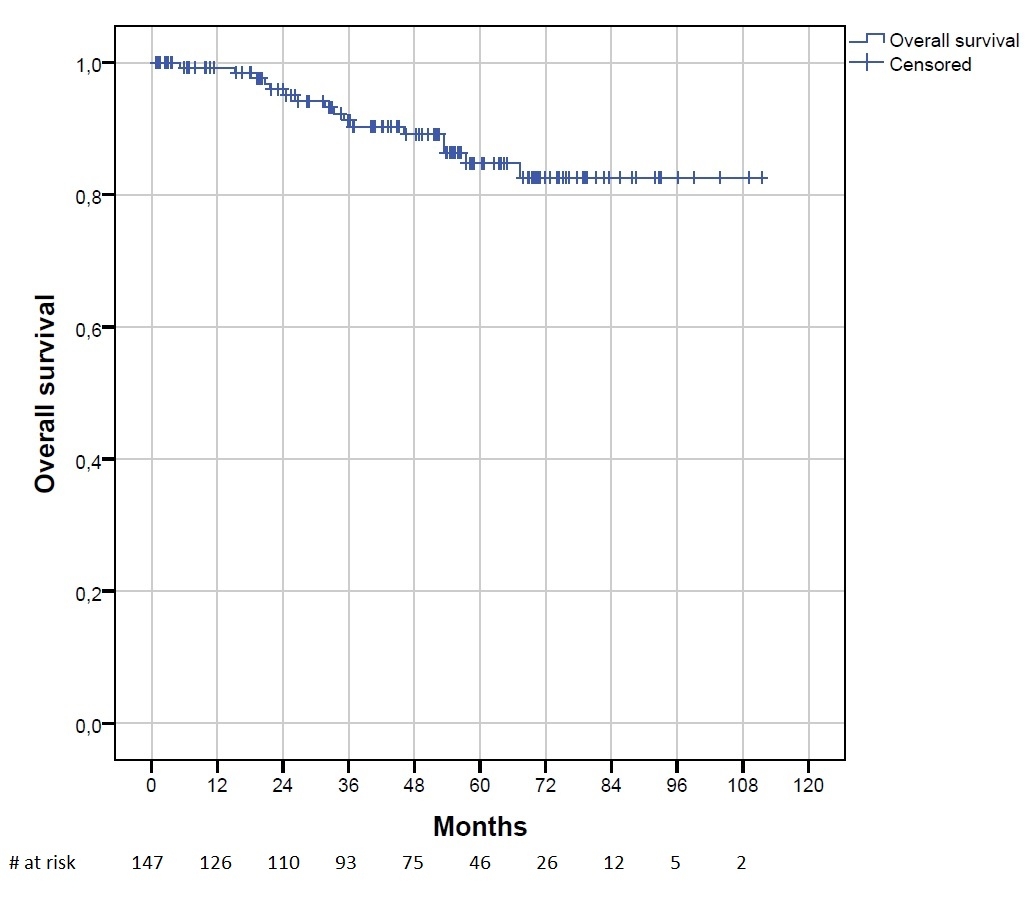

Supplement: Supplementary file 2 — Graph 3: Local control in all patients [file 66_2022_2002_MOESM2_ESM.jpg]

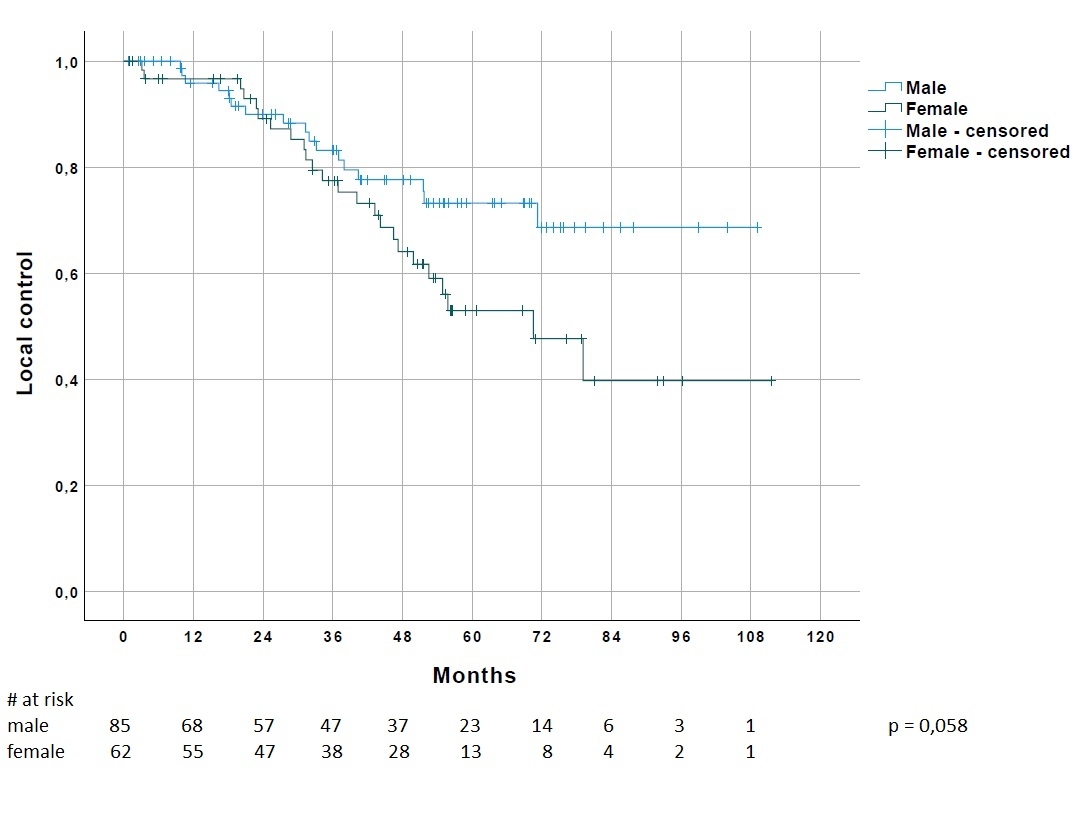

Supplement: Supplementary file 3 — Graph 5: Subgroup analysis concerning sex [file 66_2022_2002_MOESM3_ESM.jpg]

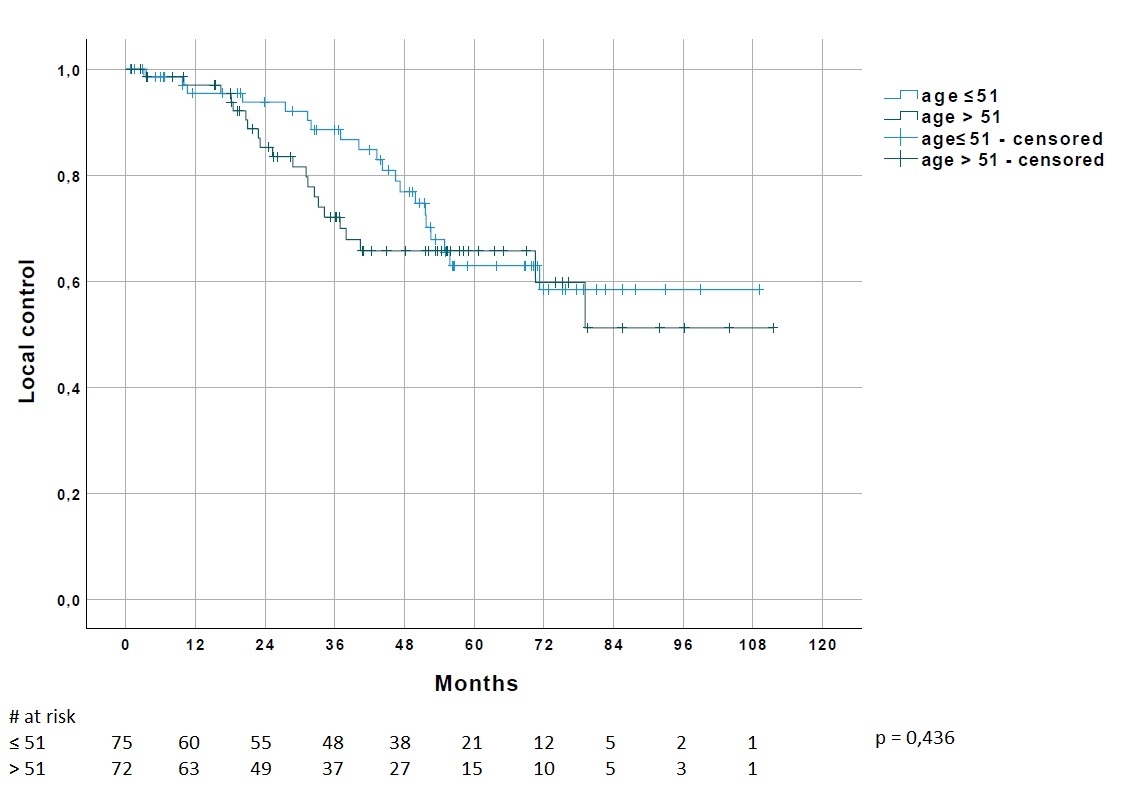

Supplement: Supplementary file 4 — Graph 6: Subgroup analysis concerning age [file 66_2022_2002_MOESM4_ESM.jpg]

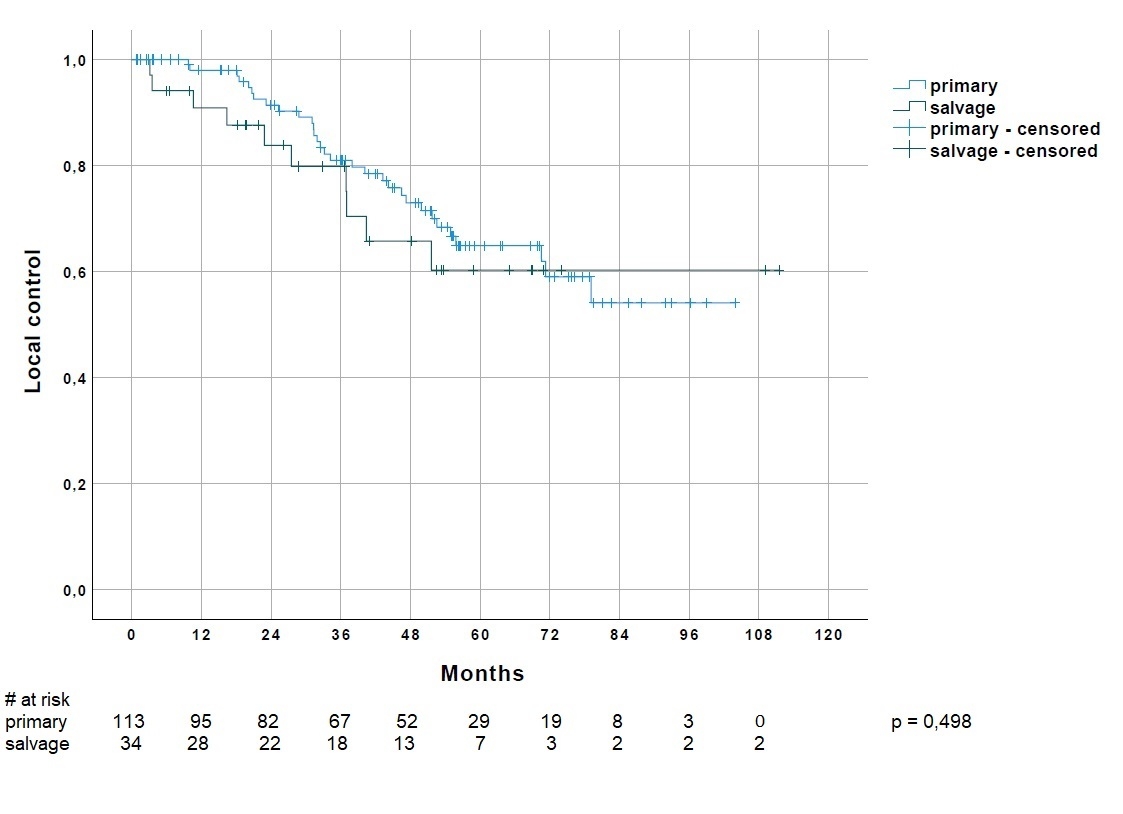

Supplement: Supplementary file 5 — Graph 7: Subgroup analysis concerning primary vs. recurrent radiotherapy [file 66_2022_2002_MOESM5_ESM.jpg]
